# Supplementary material for: DMRforPairs: identifying Differentially Methylated Regions between unique samples using array based methylation profiles
Source: BMC Bioinformatics. 2014 May 15;15:141. doi: 10.1186/1471-2105-15-141 (PMC4046028; doi:10.1186/1471-2105-15-141)
Supplement: Additional file 2 — DMRforPairs output for the comparison of A431-MCF7 and NA17018-NA17105. Please start from the HTML files in each folder. Available via the BMC Bioinformatics website. [file 1471-2105-15-141-S2.zip › 1394847754114233_MOESM2_ESM/A431_MCF7/figures/13057.pdf]

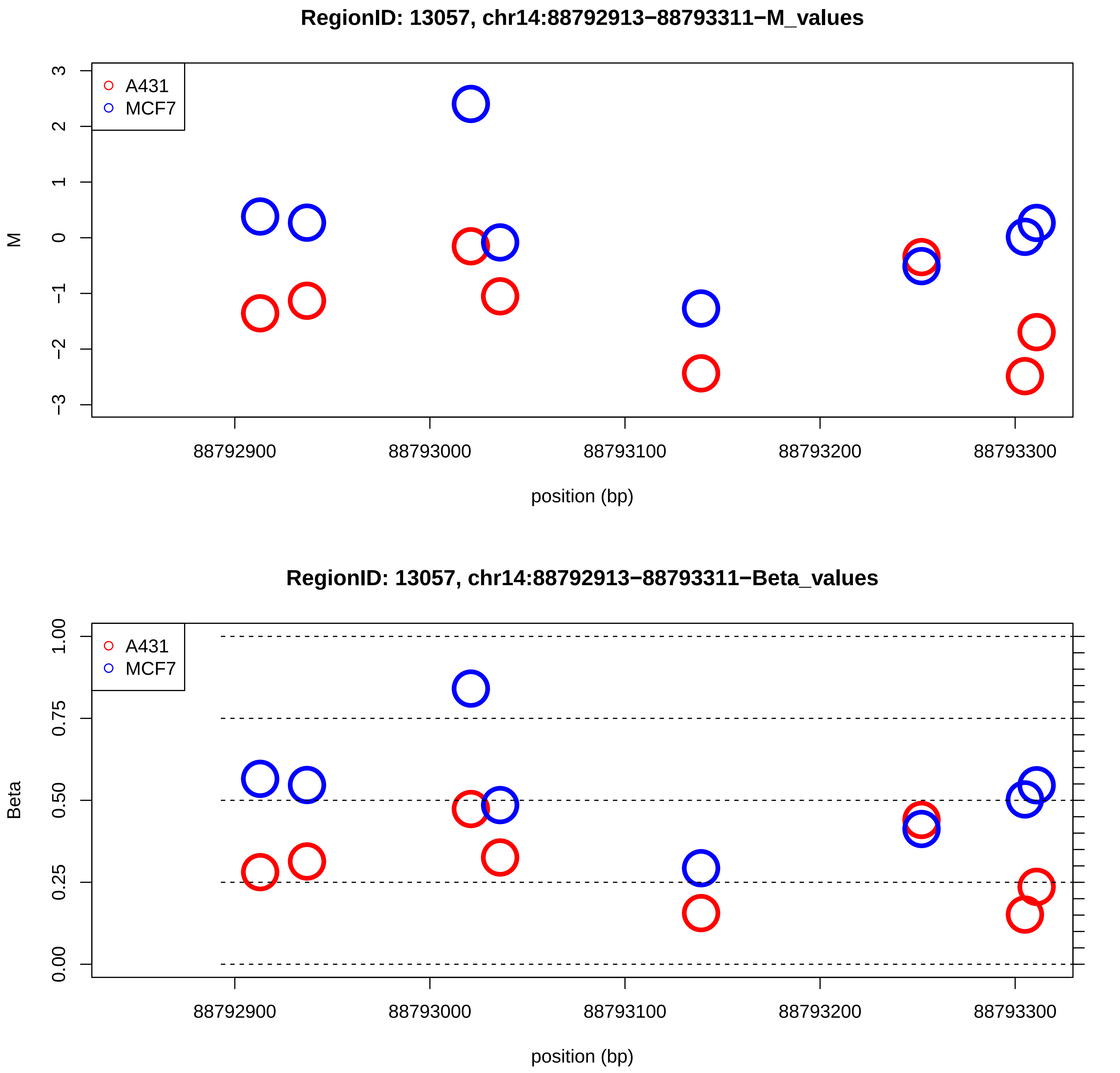
RegionID: 13057, chr14:88792913−88793311−M_values
A431
MCF7
M
3
2
1
0
−1
−2
−3
88792900
88793000
88793100
88793200
88793300
position (bp)
RegionID: 13057, chr14:88792913−88793311−Beta_values
A431
MCF7
Beta
1.00
0.75
0.50
0.25
0.00
88792900
88793000
88793100
88793200
88793300
position (bp)

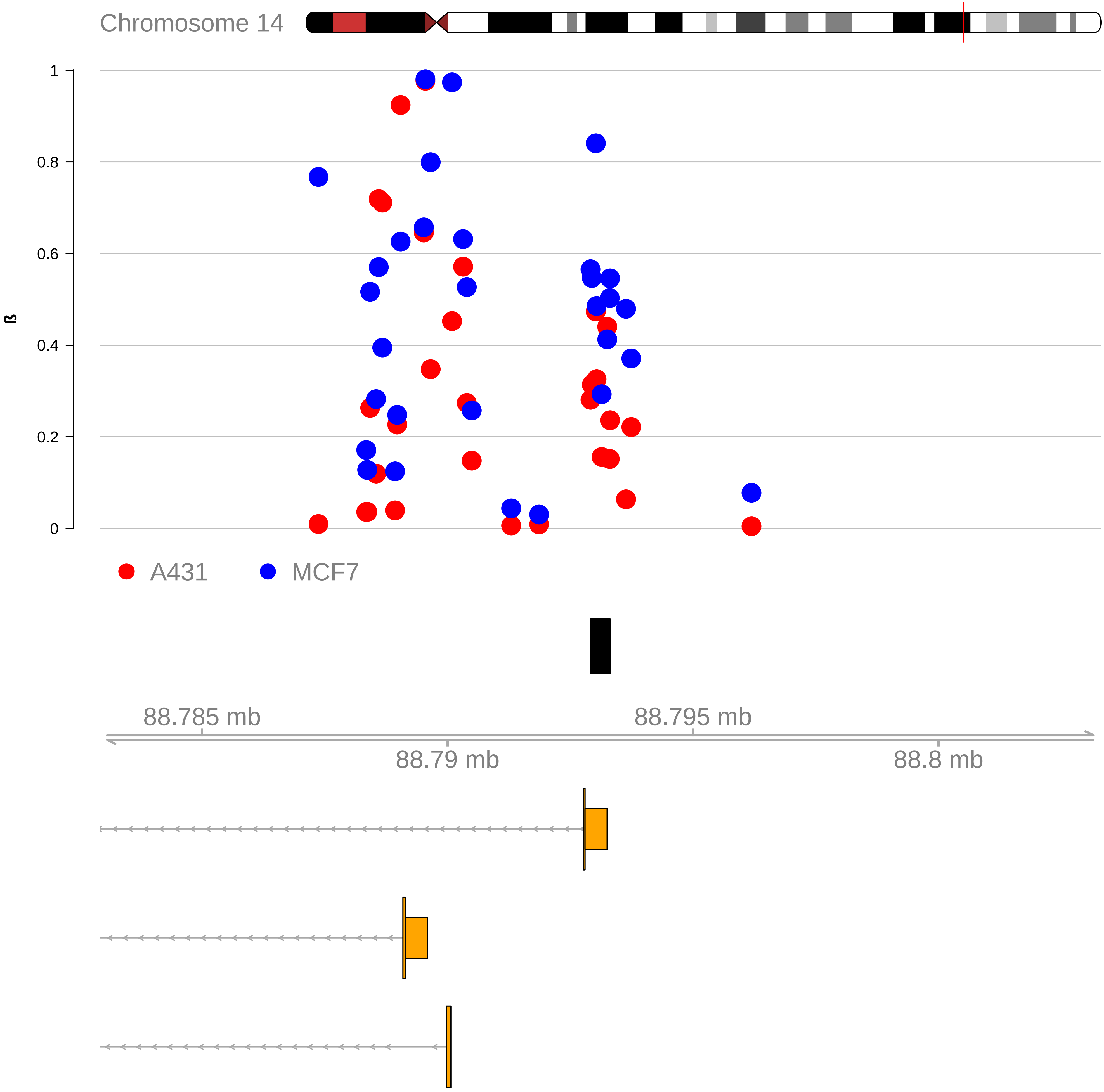
Chromosome 14
1
0.8
0.6
0.4
0.2
0
ß
A431
MCF7
88.785 mb
88.795 mb
88.79 mb
88.8 mb
